# Selection and Evaluation of a Thornless and HLB-Tolerant Bud-Sport of Pummelo Citrus With an Emphasis on Molecular Mechanisms

**DOI:** 10.3389/fpls.2021.739108

**Published:** 2021-08-31

**Authors:** Bo Wu, Na Li, Zhanao Deng, Feng Luo, Yongping Duan

**Affiliations:** ^1^School of Computing, Clemson University, Clemson, SC, United States; ^2^United States Department of Agriculture-Agriculture Research Service-United States Horticultural Research Laboratory, Fort Pierce, FL, United States; ^3^College of Horticulture, Hunan Agricultural University, Changsha, China; ^4^Department of Environmental Horticulture, Gulf Coast Research and Education Center, IFAS, University of Florida, Wimauma, FL, United States

**Keywords:** pummelo, bud sport, transcriptome, thornless, huanglongbing, alternative splicing, allelic expression difference

## Abstract

The selection of elite bud-sports is an important breeding approach in horticulture. We discovered and evaluated a thornless pummelo bud-sport (TL) that grew more vigorously and was more tolerant to Huanglongbing (HLB) than the thorny wild type (W). To reveal the underlying molecular mechanisms, we carried out whole-genome sequencing of W, and transcriptome comparisons of W, TL, and partially recovered thorny “mutants” (T). The results showed W, TL, and T varied in gene expression, allelic expression, and alternative splicing. Most genes/pathways with significantly altered expression in TL compared to W remained similarly altered in T. Pathway and gene ontology enrichment analysis revealed that the expression of multiple pathways, including photosynthesis and cell wall biosynthesis, was altered among the three genotypes. Remarkably, two polar auxin transporter genes, PIN7 and LAX3, were expressed at a significantly lower level in TL than in both W and T, implying alternation of polar auxin transport in TL may be responsible for the vigorous growth and thornless phenotype. Furthermore, 131 and 68 plant defense-related genes were significantly upregulated and downregulated, respectively, in TL and T compared with W. These genes may be involved in enhanced salicylic acid (SA) dependent defense and repression of defense inducing callose deposition and programmed cell death. Overall, these results indicated that the phenotype changes of the TL bud-sport were associated with tremendous transcriptome alterations, providing new clues and targets for breeding and gene editing for citrus improvement.

## Introduction

A bud-sport is a phenotypically distinct part of a plant, frequently observed in woody perennials (Foster and Aranzana, 2018). Bud-sports usually harbor a limited number of mutations based on the original plants and retain most of the original traits, making them an excellent resource for breeding new cultivars. Many bud-sports have been developed into new cultivars in several important horticultural plants such as citrus (Usman and Fatima, 2018), apple (Li et al., 2018), and grape (Xu et al., 2019). In *Citrus*, which has a relatively long juvenile period, the selection of bud-sports has been widely applied in breeding. The most notable instance is the sweet orange [*Citrus sinensis* (L.) Osbeck], in which more than one hundred cultivars have been selected from bud-sports. Many cultivars in another economically important cultivar group, grapefruit (*Citrus* × *paradise* Macfayden), were also derived from bud-sport selections (Uzun et al., 2010).

Bud-sports are a valuable source of new cultivars and critical materials for studying molecular mechanisms underlying essential traits. A study on Sicilian blood oranges (*C. sinensis*) revealed that the translocation of a retrotransposon affected the expression of an anthocyanin production activator-encoding gene, *Ruby*, and caused the accumulation of anthocyanin in the fruit (Butelli et al., 2012). Transcriptome profiling has been widely used to identify the differentially expressed genes (DEGs) between citrus bud sports and their corresponding wild types. Studies on late-ripening mutants from sweet orange, clementine (*Citrus* × *clementina* Hort. ex Tan.), and “Wuzishatangju” (*C. reticulata* L.) identified tens to hundreds of DEGs at different developing stages, respectively (Wu J. et al., 2014; Wang J. et al., 2017; Wang L. et al., 2017; Terol et al., 2019). Similar studies have also been conducted on an orange-pericarp pummelo mutant (Guo et al., 2015), a wax deficient sweet orange mutant (He et al., 2018), a sweet orange mutant with impaired carotenoid biosynthesis (Romero et al., 2019), two red flesh sweet orange mutants (Pan et al., 2012; Yu et al., 2012), and two sweet orange mutants different in citrate content (Lu et al., 2016).

Pummelo [*C. maxima* (J. Burman) Merrill] is one of the economically important species in *Citrus*. Like most *Citrus* species, pummelo has thorny branches during the juvenile stage, which are believed to protect them from herbivores. In contrast, new branches grown from mature trees usually have no or much fewer and shorter thorns. Different early flowering transgenic plants could be thorny or thornless (Peña et al., 2001; Velázquez et al., 2016), indicating the pathways controlling the thorn development and the juvenile period in citrus are only partially overlapped. We also observed that some 4-year-old seedlings of sour orange (*Citrus aurantium* L.) bearing fruits remained thorny. In contrast, its thornless mutant and other thorny sibling plants remained in the juvenile state. Thorns are supposed to be modified lateral branches or axillary buds (Singh, 2019), whose development is related to the crosstalk between cytokinin and auxin (Schaller et al., 2015) and other plant hormones. Through silencing two TCP transcription factor genes, TI1 and TI2, citrus thorns were successfully transformed into branches (Zhang et al., 2020). In this study, the thorns were repressed rather than converted on thornless bud-sport (TL) branches.

When compared to the original plant (W), TL and its partially recovered mutants with short thorns (T) showed enhanced tolerance to Huanglongbing (HLB), a devastating disease of citrus worldwide. HLB has a relatively short-recorded history of fewer than 200 years (Killiny et al., 2018), and so far, no HLB-resistant citrus cultivar is commercially available. However, some citrus species/varieties did display different degrees of tolerance (Manjunath et al., 2008; da Graça et al., 2016) and higher expression levels of basal defense-related genes (Wang et al., 2016), and typical plant defense responses, such as callose deposition, were observed in Las-infected citrus leaves (da Graça et al., 2016; Wang N. et al., 2017). Many efforts have been carried out to breed HLB resistant/tolerant citrus cultivars both by the citrus industry and scientific communities (Wang, 2019), and the selection of HLB tolerant citrus is considered an important approach. Moreover, a comparison of bud-sports with enhanced HLB tolerance/resistance with their maternal lines will unravel the molecular mechanisms of HLB tolerance/resistance in citrus.

In this study, to unravel the underlying molecular mechanisms of the phenotypical differences among the three pummelo genotypes (W, TL, and T), we compared their gene/pathway expression, alternative splicing, and allelic expression through transcriptome profiling and genotyping by genome sequencing. Several genes putatively underlying the thornless mutation were identified, and their expression was quantified in similar bud-sports from two other *Citrus* species, grapefruit and sour orange.

## Materials and Methods

### Plant Materials

A relatively tolerant seedling of *C. maxima* (J. Burman) var. “Mato Buntan” (accession no. PI 5359398) was selected via inoculation by hot psyllid that carried high titers of *Candidatus* Liberibacter asiaticus (Las) in the USHRL greenhouse, Fort Pierce, FL. The infected plant displayed asymptomatic to very mild symptoms though it carried high Las titers with Ct = 24.5. Branches from the HLB tolerant seedling (wild type, W) were grafted on sour orange rootstocks after treatment with ampicillin and streptomycin, as described by Zhang et al. (2012). The plants were maintained in the greenhouse, and a thornless bud-sport was observed 18 months after grafting. The thornless bud-sport was further propagated using bud-grafting, from which both thornless plants (TL) and partial thorn-recovery plants (T) were obtained. We also selected two thornless bud-sports from a 3-year-old Duncan grapefruit (*Citrus* × *paradisi* Macfad. var. Duncan) seedling and a common sour orange (*Citrus* × *aurantium* L. var. Amara Engl.) seedling with relatively higher HLB tolerance than their mother plants. The seedlings of these two mutants were surveyed for gene expression confirmation via qRT-PCR.

### Evaluation of HLB Resistance/Tolerance

Clones of W, T, and TL were evaluated for HLB resistance/tolerance via graft inoculation using an aggressive Las isolate in an insect-proof greenhouse. The Las titer was quantified with qPCR using the 16S rDNA primer and probe (Li et al., 2006). Las quantification by qPCR was performed in triplicate for each propagated plant and repeated every 6 months since inoculation.

### RNA Extraction and Transcriptome Sequencing

Newly expanded leaves from 3 W, 3 TL, and 4 T clonal plants were subjected to RNA extraction and RNA-seq. Another two transcriptomes (WT and TT) were sequenced using RNA extracted from thorns of W, and T. Total RNA was extracted from the leaves or thorn barks using the RNeasy Plant Mini Kit (Qiagen Inc., Valencia, CA) following the manual. The RNA concentration and quality were assessed by NanoDrop ND-1000 spectrophotometer, and concentration ≥ 250 ng/μL, OD260/OD280 = 1.8∼2.2, and OD260/OD230 ≥ 2.0 were required. More than 20 μg RNA of each sample was sent to BGI (Beijing Genomics Institute) Genomics (Shenzhen, China), which carried out poly-A selection of mRNA, pair-end library construction, and high-throughput sequencing on HiSeq2000 (Illumina, San Diego, CA) sequencing machine. More than 5 Gb sequencing reads were obtained for each sample.

### Differential Expression Analysis on the Transcript and Gene Levels

The quality of sequencing reads was examined by FastQC v0.11.8,^1^ after which Trimmomatic v0.38 was applied in read cleaning (Bolger et al., 2014). Adapters and low-quality read ends (average base quality < 15) were trimmed from reads. Sequencing reads including ≥ 5% ambiguous bases (N) were discarded.

A strategy based on both assembly and mapping (Pertea et al., 2016) was used to enhance the gene structure annotation of the pummelo reference genome (Wang X. et al., 2017). The reads from the 12 transcriptomes were mapped to the same haploid pummelo genome used in the variant analysis by HISAT2 v2.1.0 (Kim et al., 2015). The obtained RNA-seq alignments were further assembled into potential transcripts using StringTie v1.3.4 (Pertea et al., 2015), and gene structures were inferred from all the 10 assemblies, which were merged with the gene structure annotation of the pummelo reference (Wang X. et al., 2017) by StringTie v1.3.4 to generate one merged gene structure annotation. Statistics on novel genes and transcripts were carried out by comparing the merged gene structure annotation with the reference gene structure annotation using gffcompare in StringTie v1.3.4. The longest transcripts of the novel genes were further aligned to the transcript sequences annotated in the *Citrus sinensis* (Xu et al., 2013) and *Citrus clementina* (Wu G. A. et al., 2014) reference genomes via blastn. Genes or transcripts with targets sharing ≥ 95% nucleotide similarity, ≥ 50% alignment coverage of the queries, and *E*-value ≤ 1E-3 were recognized as annotated in the reference genomes.

Based on the merged annotation, the transcriptomes were compared pairwise among W, TL, and T at the gene and transcript (gene isoform) levels. The expression of transcripts was estimated by StringTie v1.3.4 using the acquired RNA-seq alignments. Data normalization and statistical analysis on transcript levels were processed by R package ballgown v3 (Frazee et al., 2015). For gene-level analysis, the raw read counts per gene were calculated using Salmon v0.11.3 (Patro et al., 2017) and Tximport v1.12.1 (Soneson et al., 2015). Gene read count was normalized using the median of ratios method (Love et al., 2014). The Wald test for the generalized linear model (GLM) coefficients was applied in the differential expression test using DESeq2 v1.28.0 (Love et al., 2014).

Gene expression correlation analysis, principal component analysis (PCA), and hierarchical clustering were carried out among the 12 transcriptomes using Pivot v1.0.0 (Zhu et al., 2018). In hierarchical clustering, Euclidean distance was calculated based on log10 (Normalized gene read counts), and the Ward.D2 method was applied in agglomeration.

### Allelic Expression Difference (AED) Analysis

Long-read sequencing of whole-genome DNA was applied to detect exonic heterozygous variants in W. The genomic DNAs were extracted from the leaves of W using the DNeasy Plant Mini Kit (Qiagen Inc., Valencia, CA). The quality of the DNAs was measured using NanoDrop ND-1000 spectrophotometer (Thermo Fisher Scientific, Waltham, MA) and agarose gel electrophoresis. Library construction and sequencing on the PacBio RS II system (Pacific Biosciences, Menlo Park, CA) was carried out by Yale Center for Genome Analysis (YCGA, CT, United States).

Two different methods were applied to identify SNVs (Single nucleotide variants) based on the sequencing reads. The published haploid pummelo genome was used as a reference in both methods. We first used the Resequencing Application implemented in SMRT Analysis v6.0.0 (Pacific Biosciences, Menlo Park, CA) for variation identification. Pbalign v0.4.1 was used for mapping the reads to the reference genome, and then arrow v2.3.3 was applied to call both heterozygous and homozygous variants with the option “diploid.” In the second method, the subreads were transformed into fastq by SMRT Analysis v6.0.0 and mapped to the reference by Minimap2 v2.17 using Pacbio read mode (Li, 2018). Variant calling was performed by the UnifiedGenotyper algorithm in GATK v3.8 (McKenna et al., 2010), using the parameters suggested by Carneiro et al. (2012). Then, variants detected by both methods were identified as high-quality variants for further analysis. Based on the acquired gene structure annotation, heterozygous exonic SNVs were output by Bedtools 2.28 (Quinlan and Hall, 2010) and applied in allelic expression analysis.

Based on the BAM files from mapping RNA-seq reads to the reference in the previous step, allelic read counts were output on all exonic SNVs using the ASEReadCounter command in GATK 4.1.2.0 (McKenna et al., 2010). The reference allelic expression ratio was calculated by dividing the reference allelic count on an SNV by the total local read count (the sum of reference and alternative allele read counts). Repeated *G*-tests of Goodness-of-Fit test^2^ were carried out to check the alleles on each SNV were expressed at the same ratios within each group and among different groups (W, TL, and T). When both pooled *G*-test *p*-value and total *G*-test *p*-value were ≤ 0.001, the allelic expression was considered significantly different.

### Enrichment Analysis of Differentially Expressed Genes on Metabolic Pathways and Functional Categories

Gene Ontology annotation of all the expressed genes was carried out by Blast2GO v5.2.1 (Götz et al., 2008). Fisher’s exact test was applied to test for significantly (FDR < 0.05) enriched GO terms in biological process, molecular function, and cellular component. The general enriched GO terms were removed using Blast2GO v5.2.1 if a more specific term existed.

Gene set enrichment analysis (GSEA) was carried out using GSEA v4.0.3 (Subramanian et al., 2007). We obtained the gene sets from KEGG pathways, the WikiPathway database, and the PMN database (Schläpfer et al., 2017). The orthologs of all pummelo genes were identified in *Arabidopsis thaliana* proteins (Lamesch et al., 2012) and *Citrus clementina* genes (Wu G. A. et al., 2014) through searching for the best hit using blastx or blastn, and ≥ 50% alignment coverage and ≤ 1E-3 *E*-value were required.

Corresponding protein sequences of all plant disease resistance genes (R genes) were downloaded from PRGdb 3.0 database (Osuna-Cruz et al., 2018). All pummelo genes were searched against the database using blastx, and any gene that had a blastx hit with E-value ≤ 1E-3 and ≥ 50% coverage of the target was considered as a putative R gene. The putative R genes in our gene set were output in FASTA format and submitted to Drago 2 server (Osuna-Cruz et al., 2018), which searched the leucine-rich region, kinase domain, nucleotide-binding region, Toll-interleukin region, coiled-coil, and transmembrane domain with HMM modules. Based on the results, the R genes were classified according to the domain combinations (Osuna-Cruz et al., 2018). Other plant defense response genes were obtained through their GO annotation.

### RT-qPCR Quantification of Gene Expression

cDNA synthesis was performed with oligo (dT) primers using the M-MLV reverse transcriptase system (Promega Corporation, Madison, United States) according to the manufacture’s instructions. A 20 μL reaction was applied in qPCR, including 10 μL of 2 × FAST SYBR Green Master Mix (Quanta Bio) reagent and 2 μL of DNA template. The following standard thermal profile was used for all amplifications: 95°C for 5 min, followed by 40 cycles of 95°C for 3 s and 60°C for 30 s. Primer sequences are listed in Supplementary Table 1, and ACT2 and GAPDH primers from the study of Mafra et al. (2012) were used as reference genes. Reactions were performed in triplicate, and the 2^–ΔΔ^ ^Ct^ method was used to calculate relative expression as described by Pitino et al. (2015).

## Results

### Phenotype Difference Among the Analyzed Pummelo Genotypes

**FIGURE 1:**
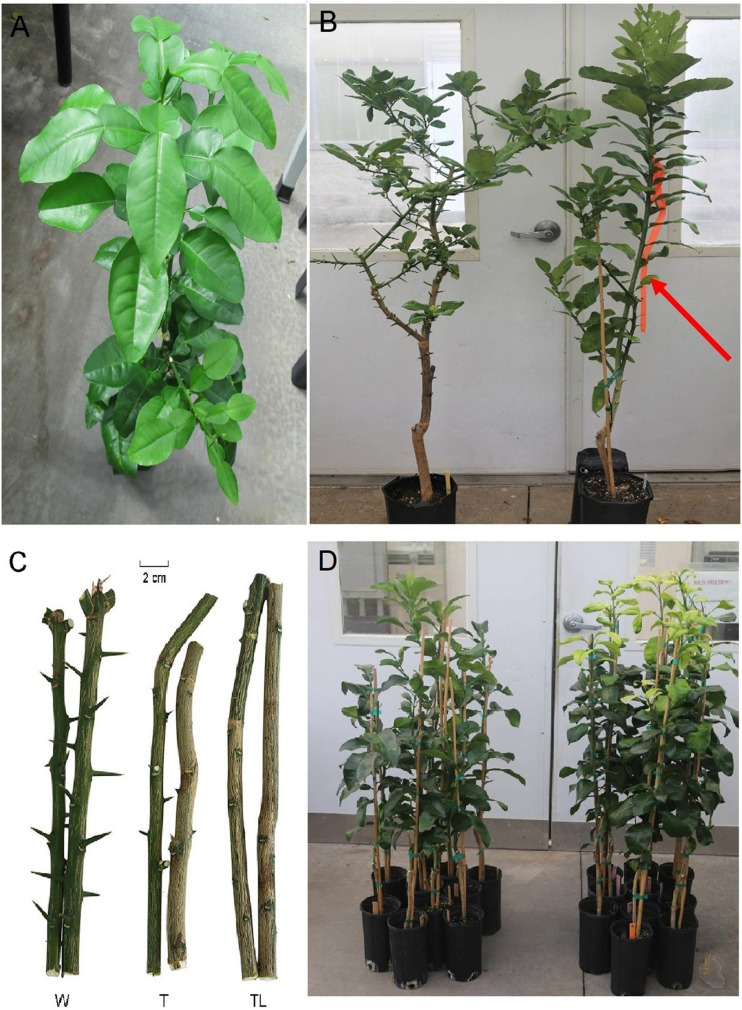
Pummelo thornless bud-sport selection and evaluation. **(A)** Asymptomatic HLB-tolerant Mato Buntan seedling (wild type W) with CT = 24.5 1 year after Las inoculation. **(B)** Comparison of the growth of W (left) and thornless bud-sport (red arrow). **(C)** The branches of W, T, and TL. **(D)** The HLB-tolerant mutants (TL and T) in the left show growth retardation, while the susceptible grapefruit plants on the right showed typical yellow shoot symptoms 12 months after inoculation with an aggressive Las isolate.

Among the three analyzed genotypes, the thornless genotype (TL) was obtained from a bud mutation of the original seedling (wild type, W) in Figure 1A. The partially recovered thorny genotype (T) was derived from the bud propagations of TL with a ∼50% ratio (14 of 30). The difference in phenotype was mainly found in two aspects among W, T, and TL (Figures 1B,C). The first is the thorn length. W and T branches are thorny, while the TL plants are thornless. However, there was a significant difference in the thorn length between W and T. The latter had a 65% reduction compared with the wild type. The second difference is in the growing vigor of the bud-sport. As shown in Figure 1B, T and TL grew more vigorously than W. All the three genotypes have shown high HLB tolerance in our tests compared with the other original pummelo sibling seedlings. In our HLB tolerance/resistance tests, at 6 months after inoculation with a highly virulent Las isolate, no typical HLB symptom but only slight growth retardation was observed on TL and T (Figure 1D). However, on the tested non-tolerant grapefruit and W seedlings, severe symptoms (yellow shoots) and mild symptoms were observed, respectively. The RT-PCR results showed that the titers of Las bacteria were not significantly different between W and T or TL, but were significantly lower (*p* < 0.05 by *t*-test) than those in grapefruit.

### Novel Transcript and Gene Discovery From RNA-Seq Data

We carried out RNA-seq on 10 leaf samples from 3 W, 3 TL, and 4 T clonal plants and two thorn samples (WT and TT) from W and T. An assembly-based strategy was applied to discover novel genes and transcripts absent in the reference annotation. 4,165 putative novel gene loci and 38,825 putative novel transcripts were identified from the 12 transcriptomes (Supplementary Table 2), which increased the number of analyzed genes and transcripts by 14.1 and 89.2% compared to the pummelo reference (Wang X. et al., 2017) gene models. Of the 4,165 putative novel gene loci, 1,133 were present in the gene annotation of *Citrus sinensis* (Xu et al., 2013) or *Citrus clementina* (Wu G. A. et al., 2014). Among the rest 3,032 loci, 1,303 had homologous proteins in the NCBI nr database, while the remaining 1,729 could be genes specific in *Citrus* (*maxima*) or non-coding RNAs. On average, each expressed gene had 1.14 novel transcripts detected in this study. Among the novel transcripts, 33,423 belonged to genes annotated in the reference genome, and the rest 4,830 belonged to the 4,165 novel gene loci.

### Transcriptome Profiling and Clustering

**FIGURE 2:**
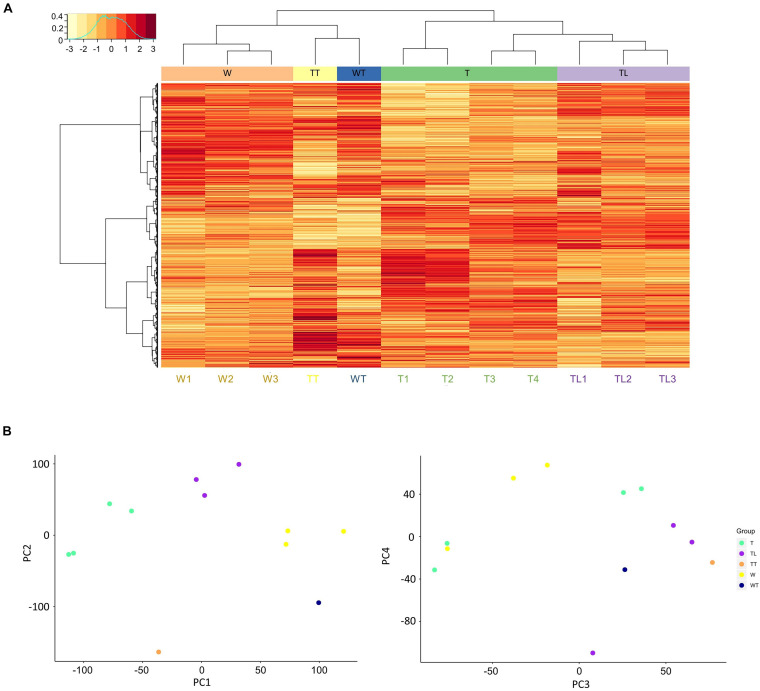
Clustering of the transcriptomes. **(A)** Hierarchical clustering and gene expression heatmap. In the heat map, each row represents a gene, and each column represents a transcriptome. The clustering trees based on transcriptomes and genes are shown on the top and to the left of the heat map. In the left top legend, the horizontal axis denotes the normalized expression values of genes, and the vertical axis shows the normalized frequency of genes at the different expression levels. **(B)** Distribution of samples along PC1 to 4 from the principal component analysis.

Gene expression correlation analysis showed that intra-group transcriptomes generally had a higher Pearson correlation coefficient than inter-group transcriptomes, except for the T leaf transcriptomes. As shown in Supplementary Figure 1, T3 and T4 had higher correlation coefficients with most TL transcriptomes than T1 and T2. The W and TL leaf transcriptomes were clustered into separate clades by hierarchical clustering (Figure 2A), and the two thorn transcriptomes (TT and WT) were clustered together. The four T leaf transcriptomes clustered with the TL clade, and both T3 and T4 were closer to TL leaf transcriptomes than T1 and T2 (Figure 2A).

In PCA, 11 principal components (PC) explain ∼100% total variability of the transcriptomes (Supplementary Figure 2). The first two components PC1 and PC2 accounted for 51.9% of the total variance. On PC1 (28.3% of total variance), the distribution of leaf transcriptomes was mainly explained by the origin relationship among W, TL, and T (Figure 2B). PC2 (23.6%) could mainly be explained by the thorn-related phenotype difference. Since the two thorn transcriptomes, WT and TT, lay below all the leaf transcriptomes, and W1-3 and T1-4 lay below TL1-3 (Figure 2B).

### Differentially Expressed Pathways in the Bud Sports

Among pairwise comparisons, 3,496 and 4,057 differentially expressed genes (DEG) were identified between W and TL and between TL and T, respectively (Supplementary Figure 3). Interestingly, the most DEGs (6,378) were detected between W and T. A majority of DEGs between TL and W (2290/3496) remained significantly upregulated or downregulated in T compared with W (Supplementary Figure 3).

**FIGURE 3:**
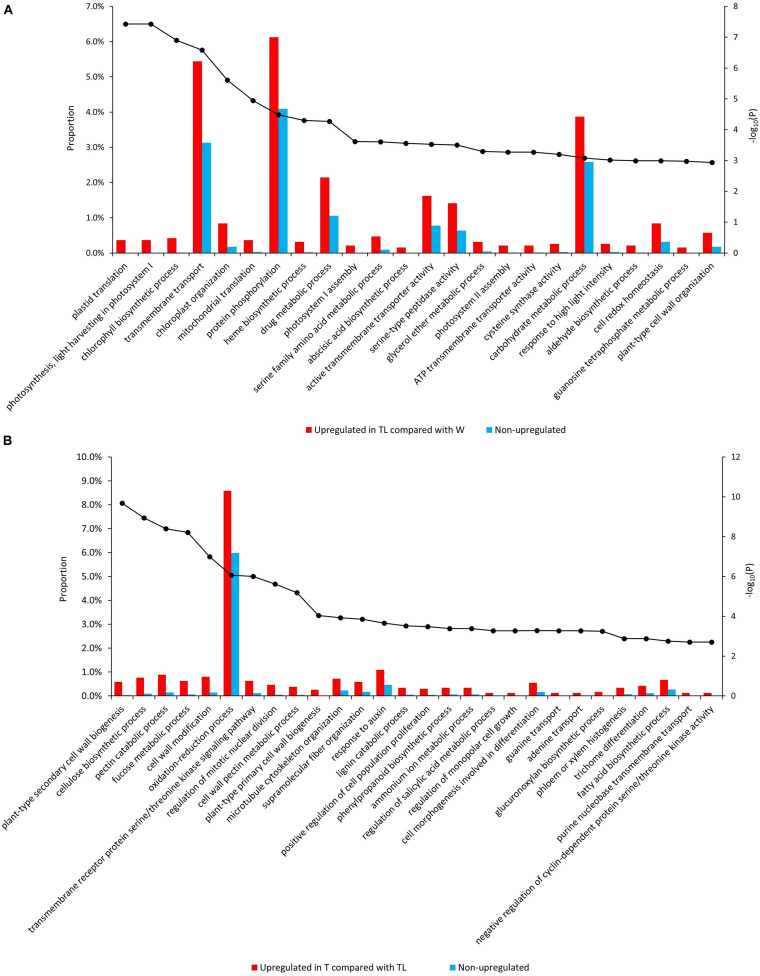
Enriched GO terms from the comparative transcriptomic analysis. Significantly enriched GOs in genes upregulated in TL compared with W **(A)** and those upregulated in T compared with TL **(B)**. The bars denote the proportion of genes (the left vertical axis) with each GO in the tested gene sets, and the black dots indicate the –log10 (*p*-value of enrichment test) (the right vertical axis) of each GO.

Gene ontology (GO) and pathway enrichment analysis revealed multiple pathways with significant expression alteration in W to TL transition. Compared with W, 204 GOs/12 pathways and 43 GOs/2 pathways were significantly (FDR < 0.05) over-represented for genes upregulated (1,995) and down-regulated (1,519) in TL, respectively (Supplementary Table 3). Multiple upregulated pathways were related to photosynthesis (Figure 3A, Supplementary Figure 4, and Supplementary Table 3). The down-regulated pathways included the cell wall biogenesis and several other pathways (Supplementary Figure 5A and Supplementary Table 3).

Compared to TL, 84 GOs/9 pathways, and 15 GOs/6 pathways were significantly over-represented for the upregulated and downregulated genes in T, respectively. Genes involved in cytokinin signaling, response to auxin, salicylic acid (SA) metabolic process regulation, anatomical structure development, cell cycle regulation, and several cell wall metabolism-related pathways were enriched in upregulated genes in T (Supplementary Table 3). Several processes down-regulated in TL (compared with W) were upregulated in T (compared with TL), including secondary cell wall biosynthesis, xyloglucan biosynthesis, carbohydrate biosynthetic process, lipid metabolism, and lignin metabolism (Figure 3B and Supplementary Figure 6). A few GO terms and pathways were also down-regulated in T compared with TL (Supplementary Figure 5B and Supplementary Table 3).

### DEGs and Pathways Putatively Responsible for the Thornless Mutation

Because both W and T are thorny while TL is thornless, common DEGs between TL and both W and T may be responsible for the thornless mutation. Accordingly, we identified 293 upregulated and 397 down-regulated DEGs in TL compared to W and T (Supplementary Table 4). The upregulated and downregulated genes were distributed in multiple functional categories (Supplementary Figure 7), and both included a response to stimulus, including phytohormones and transcription factors. The transcription factors differentially expressed in TL mainly were related to phytohormone or involved in secondary cell wall biogenesis (Supplementary Table 4). The expression levels of the genes mentioned in this section below have been shown in Supplementary Figure 8.

We identified 1 upregulated and 7 down-regulated auxin-related DEGs in TL compared to W and T (Supplementary Table 4). PIN5 (auxin efflux carrier component 5, Cs6g02450) was upregulated in TL, while two other auxin transporter genes, LAX3 (auxin transporter-like protein 3, Cg3g016080) and PIN7 (auxin efflux carrier component 7, Cs1g26700), were significantly down-regulated. The rest down-regulated genes included PTL (trihelix transcription factor, Cg2g035410), IAA16 (auxin-responsive IAA16, Cs4g17050), YUC2 (Indole-3-pyruvate monooxygenase YUC2, Cs4g15810), PLC2 (phosphoinositide phospholipase C 2, Cs7g31080), and SAUR78 (small auxin upregulated RNA 78, Cs5g15740). Five cytokinin-related genes were differentially expressed in TL compared to both W and T. Among them, CYP735A1 (cytokinin hydroxylase, Cg6g019610) was significantly upregulated in TL. LOG5 (cytokinin riboside 5′-monophosphate phosphoribohydrolase, Cs3g27020), ZOG1 (zeatin-O-glucosyltransferase, Cs7g32210), CYCD3 (Cyclin-D3-1, Cg3g020940), and AHK2 (Histidine kinase 2, Cs4g02460) were significantly down-regulated in TL.

Eleven secondary cell wall biogenesis-related genes were significantly down-regulated in TL compared with both W and T. Moreover, all the 11 genes were expressed at lower levels in leaf transcriptomes than in the thorn transcriptomes. Five of them were transcription factors, including four NAC domain-containing transcription factor genes, SND1 (secondary wall-associated NAC domain protein 1, Cs5g01350), SND2 (secondary wall-associated NAC domain protein 2, Cs2g16190), NST1 (NAC secondary wall thickening promoting factor 1, orange1.1t00561), and VND1 (vascular-related NAC-domain, Cs1g25100), and MYB42 (MYB domain-containing transcription factor 42, Cg5g008830). The remaining six genes include two fasciclin-like arabinogalactan protein-encoding genes FLA11 (Cg6g002120) and FLA12 (Cg8g020560), two UDP-glucuronate: xylan alpha-glucuronosyltransferase encoding genes GUX1 (Cs1g05500) and GUX2 (Cs3g23600), IRX9 (beta-1,4-xylosyltransferase, Cg6g002620), and CTL2 (chitinase-like protein 2 gene, Cg9g001540).

### Differentially Expressed Plant Defense Genes Among the Genotypes

A total of 3,110 plant defense-related genes, including 1,594 putative R genes, were identified in the pummelo genome through bioinformatic analysis. Since no difference in HLB tolerance was observed between TL and T and both were more tolerant than W, common DEGs in TL and T compared to W may contribute to the higher HLB tolerance. Among the defense-related genes, 221 and 365 were significantly upregulated in TL and T compared with W, respectively, and 131 were upregulated in both of them (Supplementary Table 5). Besides, 125 and 295 plant defense-related genes were down-regulated in TL and T, respectively, and 68 down-regulated in both of them compared with W (Supplementary Table 5). The expressions of the genes mentioned below in this section have been shown in Supplementary Figure 9.

Four positive regulators of SA-dependent defense were significantly upregulated in both TL and T (Yang et al., 2008; Birkenbihl et al., 2017), including WRKY70 (Cs7g29570), CDR1 (constitutive disease resistance 1, Cg6g008170), and two citrus orthologs (Cs1g23170 and Cs7g27540) of the Arabidopsis methylesterase 1 gene (MES1). Two negative regulators of SA -dependent plant defense (von Saint Paul et al., 2011; Liu et al., 2015), UGT76B (UDP-Glycosyltransferase superfamily protein UGT76B1, Cg7g000350) and WRKY33 (Cg6g009940), were significantly down-regulated in both TL and T.

There were several DEGs related to the pathogen-associated molecular pattern (PAMP)-triggered immunity (PTI) and (or) effector-triggered immunity (ETI). Ten receptor-like kinases of PTI, including six cysteine-rich receptor-like protein genes (CRKs) and four L-type lectin-domain containing receptor kinase genes (LECRKs), were significantly upregulated in both TL and T (Supplementary Table 5). Eight resistance (R) genes involved in ETI were upregulated in both TL and T (Supplementary Table 5). Several genes involved in PTI or ETI were significantly down-regulated in TL and T, including ACS6 (Cg5g002190), OST1 (Cs5g07700), and SPL6 (Cs5g12260).

Defense induced callose deposition and cell-death were most likely suppressed in TL and T. MLO (MLO-like protein gene, Cs7g27330), a suppressor of callose deposition and second oxidative burst of cell-death in plant defense response (Kusch et al., 2019), had 46.4- and 29.8-fold expression abundance of W in TL and T, respectively, and was the most highly upregulated. Several genes putatively involved in defense-induced callose deposition and programmed cell death (Takahashi et al., 2003; Shindo et al., 2012; D’Ambrosio et al., 2017; Liu et al., 2017) were significantly down-regulated in TL and T, including PLC2 (Cg8g024170), HSP90-1 (Cg5g002260), two orthologs of EP3 (Cg5g026680 and Cg5g026650), and RD21A (Cs3g23180).

### qPCR Quantification of DEGs in Other Thornless Citrus Genotypes

**FIGURE 4:**
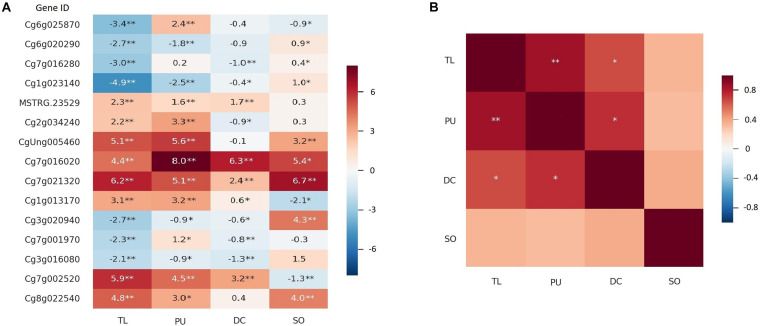
Expression of DEGs in more Citrus thornless bud-sports. The RNA-seq and qPCR results for the thornless pummelo bud-sport were shown as TL and PU, respectively. The qPCR results for Duncan grapefruit (DC) and sour orange (SO) were shown in the graphs. Single and double asterisk(s) indicate statistical significance at *p* < 0.05 and *p* < 0.001 levels, respectively. **(A)** Expression of 15 genes in bud-sports compared with their corresponding original types. The values in the cells were log2(expression value in bud-sport/expression value in original type) for RNA-seq and – ΔΔCt for qPCR. **(B)** Correlation of gene expression on the 15 genes among the citrus genotypes.

We also identified two thornless mutants with relatively higher HLB tolerance in another two Citrus species, grapefruit (*C.* × *paradisi* var. “Duncan”) and sour orange (*C.* × *aurantium* L.) (Supplementary Figure 10). qPCR quantification was executed on 15 candidate DEGs between W and TL in all the selected lineages. As shown in Figure 4A, 12 of the 15 genes showed consistent upregulated and downregulated patterns between RNA-seq and qPCR analyses for TL. The gene expression changes in thornless Duncan grapefruit (DC) were highly similar and significantly correlated with those in TL, while sour orange (SO) was more different (Figure 4).

Genes with similar expression alterations in different thornless mutants were probably related to thorn development or higher HLB tolerance. Two genes, SEP2 (Developmental protein SEPALLATA 2, Cg7g016020) and EXPA1 (expansin-A1, Cg7g021320), were significantly upregulated in all three thornless genotypes. PYR1 (abscisic acid receptor PYR1, MSTRG.23529) and SAG21 (senescence-associated gene 21, Cg1g013170) were significantly upregulated in TL, PU, and DC, while LAX3 (auxin transporter-like protein 3, Cg3g016080) and CYCD3 (CYCLIN D3, Cg3g020940) were significantly down-regulated in them. A SAUR-like auxin-responsive protein family gene (CgUng005460) and EXPA4 (expansin A4, Cg8g022540) were both upregulated in TL, PU (qPCR results for TL), and SO.

### Change in Alternative Splicing in Bud-Sports

Gene alternative splicing was widely observed in the transcriptomes. At least two transcripts were detected on 48.1–49.2% of the expressed genes in the transcriptomes (Supplementary Figure 11). The proportions of genes with different numbers of transcripts detected were highly similar among the transcriptomes.

*G*-test of independence was performed to test the hypothesis if the transcript expression ratio was consistent within each group. The null hypothesis was rejected (*p* < 0.001) for 85.2—87.5% of transcripts within each of the three leaf-transcriptome groups, TL, W, and T (Supplementary Figure 11). The test was also carried out between the two thorn transcriptomes, WT and TT, and the null hypothesis was rejected for the majority (67.0%) tested transcripts. These results suggested that the expression ratios of most transcripts were distributed in a specific range rather than at a fixed ratio.

**FIGURE 5:**
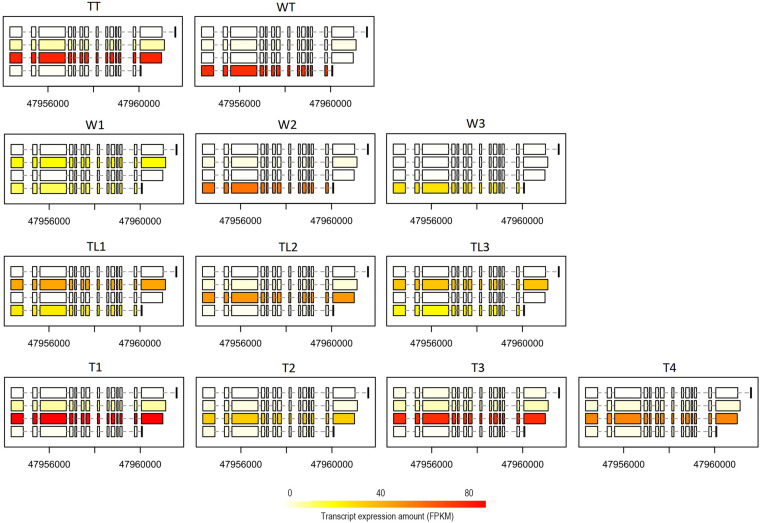
Expression of ARF6 at the transcript level in the 12 transcriptomes. For each transcriptome, the expression quantities of the four different transcript forms have been indicated by the color depth of the rectangles (exons) connected by dashed lines (introns) in the box.

We carried out a pairwise comparison on transcript expression ratio among W, TL, and T. The results showed that the expression ratio of the transcript was significantly different (*p* < 0.001) for 77 (belonging to 64 genes), 70 (58), and 178 (146) transcripts between W and TL, T and TL, and T and W, respectively (Supplementary Table 6). For instance, on ARF6 (auxin response factor 6, Cs2g09440.1), the 4th transcript had a higher expression ratio (*p* = 0.077) in W than TL, while the 3rd transcript was expressed significantly higher (*p* = 3.6e-7) in T than in W (Figure 5). The 2nd transcript of BAT1 (bidirectional amino acid transporter 1, Cs2g24220) was expressed at a significantly (*p* < 0.001) lower ratio in TL than in T, W, WT, and TT (Supplementary Figure 12).

### Allelic Expression Difference (AED) Among the Transcriptomes

Based on whole-genome sequencing of W, a total of 1,749,437 high-quality SNVs were detected, of which 1,007,731 were heterozygous, and 738,319 were homozygous. In allelic expression difference (AED) analysis, 287,115 heterozygous exonic SNVs were applied.

**FIGURE 6:**
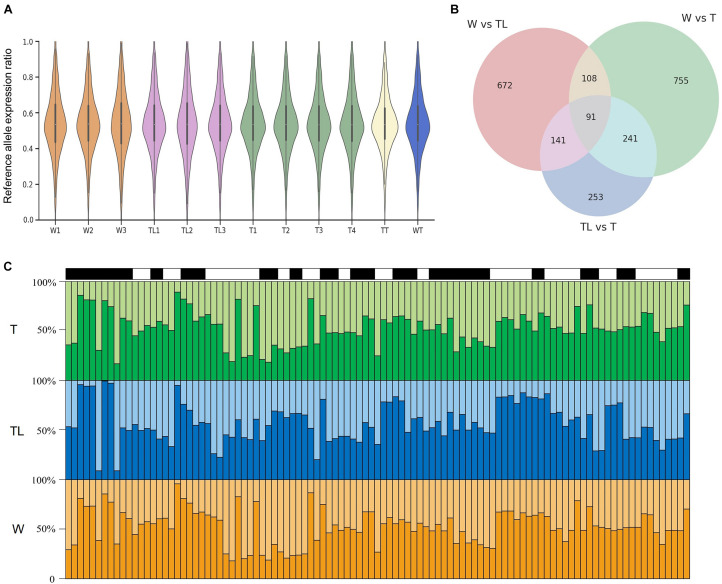
Allelic expression difference (AED) in the pummelo genotypes. **(A)** Reference allelic expression ratio distribution on heterozygous exonic SNVs in the 12 transcriptomes. **(B)** Number of SNVs with significant AED in W, TL, and T and their overlapping relationship. **(C)** Allelic expression ratio on 25 genes in which significant AED was observed between TL and both W and T. Each of the black and white rectangle represents a gene on top of the three panels, and each bin in the bar graphs represents an SNV. The vertical axis denotes the allelic expression ratio. Reference allelic expression and alternative allelic expression are represented by dark and light colors in the three panels.

The overall distribution of the reference allelic expression ratios was highly similar among the different transcriptomes (Figure 6A). The null hypotheses that both alleles were expressed at the same level were rejected (*p*< 0.001) only for a small percentage (≤ 3.2%) of the tested SNPs, suggesting that the allelic expression ratios for most SNPs were stable within each transcriptome group. Pairwise tests among the three leaf-transcriptome groups were implemented to detect inter-group AEDs. As shown in Figure 6B, AEDs (*p*< 0.001) were identified between W and TL, W and T, and TL and T for 1067, 1263, and 782 SNVs, respectively, located in 602, 647, and 433 genes. The 433 genes on which AED was observed on at least two SNVs between at least two genotypes were listed in Supplementary Table 7. For 25 genes, significant AED was observed on at least 2 SNVs between TL and both W and T (Figure 6C), and on 66 genes, it was observed between W and both TL and T (Supplementary Table 7).

## Discussion

Pummelo seedlings are derived from mono-embryonic seeds. In this study, we first identified an HLB tolerant pummelo seedling and then discovered a thornless bud sport from the grafted clonal plants of the seedling. We also found similar thornless bud sports from HLB tolerant Duncan grapefruit and sour orange seedlings, respectively, at the middle of their juvenile stage (Supplementary Figure 10). The correlation between HLB tolerance and thornless mutation may provide new clues for future selection and evaluation of a bud sport aimed at citrus improvement, including HLB tolerance/resistance. Therefore, we further conducted a comprehensive transcriptome comparison and unraveled the potential molecular mechanisms conferring the phenotypical changes of the bud sports, TL and T.

The changes in the transcriptomes of TL and T occurred in multiple aspects. This study showed that there were thousands of DEGs, hundreds of genes with differential allelic expression, and tens of genes with different alternative splicing among W, TL, and T. Allelic expression patterns have long been known to be important in the development and associated with phenotypes in parent-of-origin effects in both animals and plants (Lawson et al., 2013). They also are correlated with environmental variables and associated with some human diseases (McKean et al., 2016; Knowles et al., 2017). Alternative splicing plays an essential role in protein function/interaction and eukaryote development (Yang X. et al., 2016). This study has confirmed their wide existence between bud-sports and the original plants. Though the impact of AEDs and alternative splicing have not been revealed in citrus, they could have played essential roles in its phenotypic diversity.

Genes with full or partial recovered expression in T were the most likely responsible for the TL-specific thornless phenotype. Thorns are modified lateral branches on citrus shoots that grow from lateral buds (Zhang et al., 2020). Crosstalk between auxin and cytokinin, especially polar auxin transport, has been known to play a determinant role in lateral bud activation (Müller and Leyser, 2011), a precondition for thorn development. In this study, a few auxin- and cytokinin-related DEGs were identified between TL and both W and T, including three polar auxin transporters, PIN5, PIN7, and LAX3. PIN5 has been reported to be located at the endoplasmic reticulum and involved in intracellular auxin transport in Arabidopsis (Mravec et al., 2009). PIN7 mediates cell to cell auxin transport in Arabidopsis and is involved in auxin export during shoot branching (van Rongen et al., 2019). LAX3, which is necessary for lateral root primordia origin in Arabidopsis through regulating cell wall remodeling (Porco et al., 2016), was significantly down-regulated in TL compared with leaf and thorn transcriptomes from W and T, and it was also significantly downregulated in the thornless Duncan grapefruit. Cell wall remodeling is an important regulator of meristem morphogenesis and is involved in initiating different plant organs (Yang W. et al., 2016). In this study, cell wall biogenesis and modification-related genes/pathways were generally downregulated in TL. These results suggested the thorn bud activation on TL branches could have been disturbed by altered auxin transport and cell wall metabolism.

The high HLB tolerance in TL and T could be partially derived from the enhanced SA-dependent defense response. In citrus, SA-dependent defense is positively correlated with HLB tolerance (Oliveira et al., 2019; Zou et al., 2019), and SA-dependent systematic acquired resistance (SAR) has been suggested to improve HLB tolerance in citrus germplasms (Manjunath et al., 2008; Hu et al., 2018). Several positive regulators of SA-dependent defense have been reported to be significantly upregulated in HLB tolerant citrus accessions, including PtCDR2, PtCDR8 (Rawat et al., 2017), and CDR1 (Albrecht and Bowman, 2012). In TL and T, four positive regulators of SA-dependent defense response, including CDR1 were significantly upregulated, and two negative regulators were significantly down-regulated compared with W, implying the SA-dependent defense was enhanced.

Repression of callose deposition in the phloem could have enhanced HLB tolerance in TL and T. Though callose deposition is an important defense response in plants, it has not worked efficiently to block Las due to the slow response. Callose deposition caused by Las infection has been hypothesized to induce sieve pore blockage and starch accumulation in the leaves, and unplugging the phloem has been proposed to relieve HLB symptoms (Achor et al., 2020). Anatomical comparison between HLB susceptible and tolerant citrus accessions showed that cellulose deposition, phloem cell collapse, and starch accumulation were observed in HLB susceptible citrus, but not in an HLB-tolerant rough lemon (Fan et al., 2012). In this study, a repressor (MLO) of programmed cell death and callose deposition was significantly upregulated in both TL and T. In contrast, several genes positively related to programmed cell death and callose deposition were significantly down-regulated as shown in the results section, indicating repression of callose deposition could have contributed to their high HLB tolerance.

## Conclusion

In conclusion, we identified thornless bud-sports from pummelo, grapefruit, and sour orange seedlings in the middle of their juvenile stage. These individuals were selected as they were more HLB-tolerant in the psyllid- or graft-based evaluation. The transcriptome analyses of these thornless mutations revealed many differences in gene expression, allelic expression, and alternative splicing, though these bud-sports were derived from asexual propagation. Furthermore, the identification of thornless and HLB-tolerance-related genes may provide new clues and targets for breeding and gene editing for citrus improvement.

## Data Availability Statement

The original contributions presented in the study are publicly available. This data can be found here: National Center for Biotechnology Information (NCBI) BioProject database under accession number PRJNA557834.

## Author Contributions

YD, FL, and ZD designed the research. BW and NL drafted the manuscript. NL carried out all the lab experiments. BW carried out the bioinformatical analysis. YD selected the bud sports. All authors participated in the manuscript writing and have agreed to the published version of the manuscript.

## Conflict of Interest

The authors declare that the research was conducted in the absence of any commercial or financial relationships that could be construed as a potential conflict of interest.

## Publisher’s Note

All claims expressed in this article are solely those of the authors and do not necessarily represent those of their affiliated organizations, or those of the publisher, the editors and the reviewers. Any product that may be evaluated in this article, or claim that may be made by its manufacturer, is not guaranteed or endorsed by the publisher.

## Footnotes

1
http://www.bioinformatics.babraham.ac.uk/projects/fastqc/

2
https://rcompanion.org/rcompanion/b_09.html
