# Supplementary material for: Selection and Evaluation of a Thornless and HLB-Tolerant Bud-Sport of Pummelo Citrus With an Emphasis on Molecular Mechanisms
Source: Front Plant Sci. 2021 Aug 31;12:739108. doi: 10.3389/fpls.2021.739108 (PMC8438139; doi:10.3389/fpls.2021.739108)
Supplement: Supplementary file 2 [file Data_Sheet_2.pdf]

# Supplementary Figures

## 1 Supplementary Figures

A.

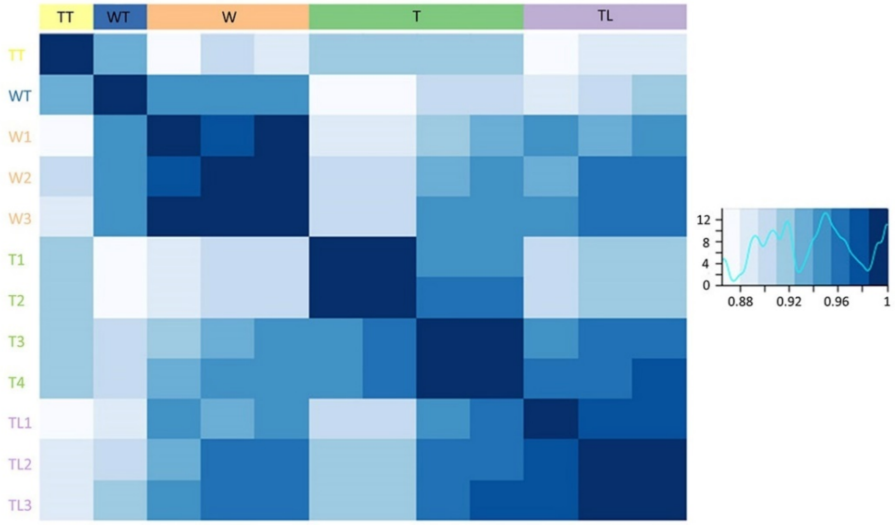


B.

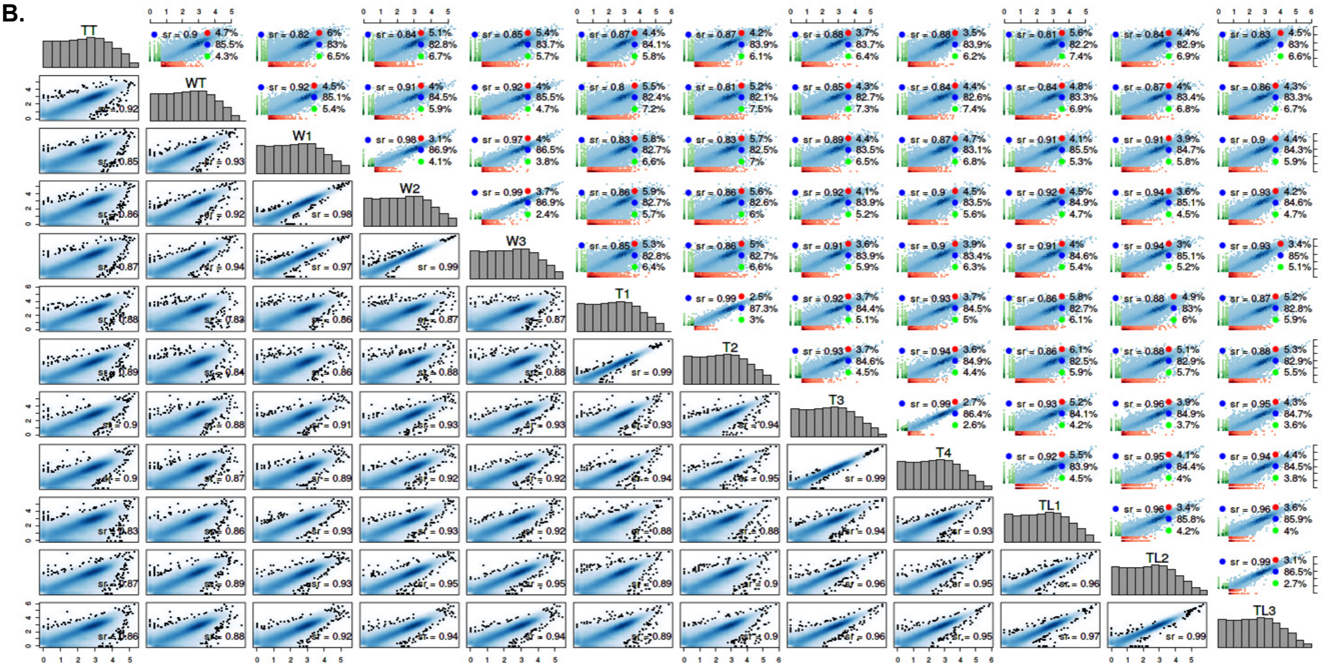


**Supplemental Figure 1. Gene expression correlation among the 12 transcriptomes.** A. Heat map of gene expression correlation coefficient. The color represents Pearson's correlation coefficient (r) in the heatmap. The horizontal axis of the legend shows the corresponding relationship between colors and the correlation coefficients. The vertical axis of the legend denotes the frequency of the

correlation coefficients. B. Pairwise comparison among the 12 transcriptomes. Each scatter plot's horizontal and vertical axis show log10(RPM) expression values of genes corresponding to a given column and row, respectively. The sr values in the plots denote the Pearson correlation coefficient r. In the smoothed scatter plots at the lower left, the black dots were the 5% genes with the most statistically different expression between the given transcriptomes. In the upper right region, blue dots denote genes detected in both transcriptomes. Green and red dots represent genes with no expression detected in transcriptomes in corresponding columns and rows.

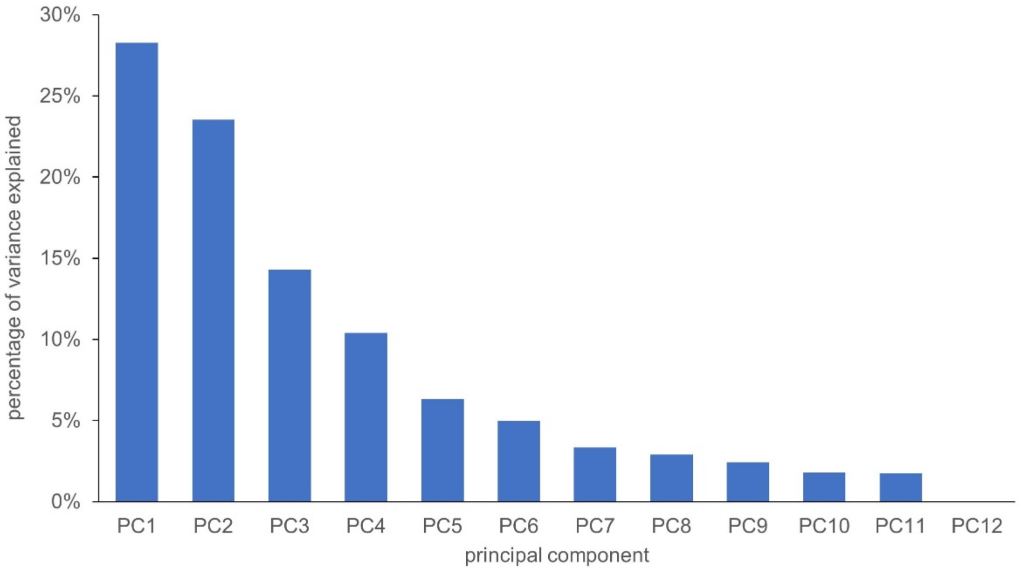


**Supplemental Figure 2. The percentage of variance each principal component could explain.**

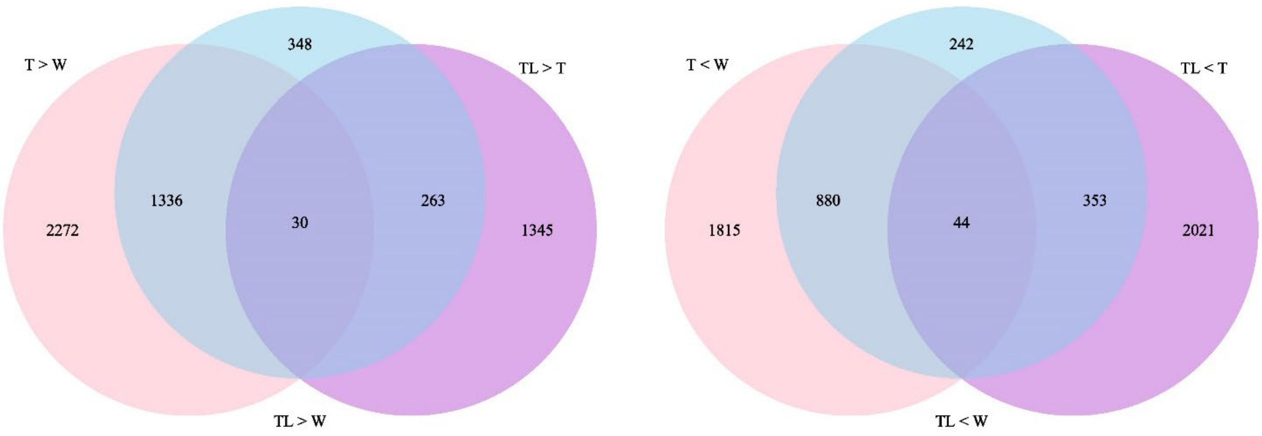


**Supplemental Figure 3. Differentially expressed genes (DEG) among leaf transcriptomes of W, TL, and T.** T > W and T < W denote genes significantly upregulated and downregulated in T compared with W, respectively, and so on.

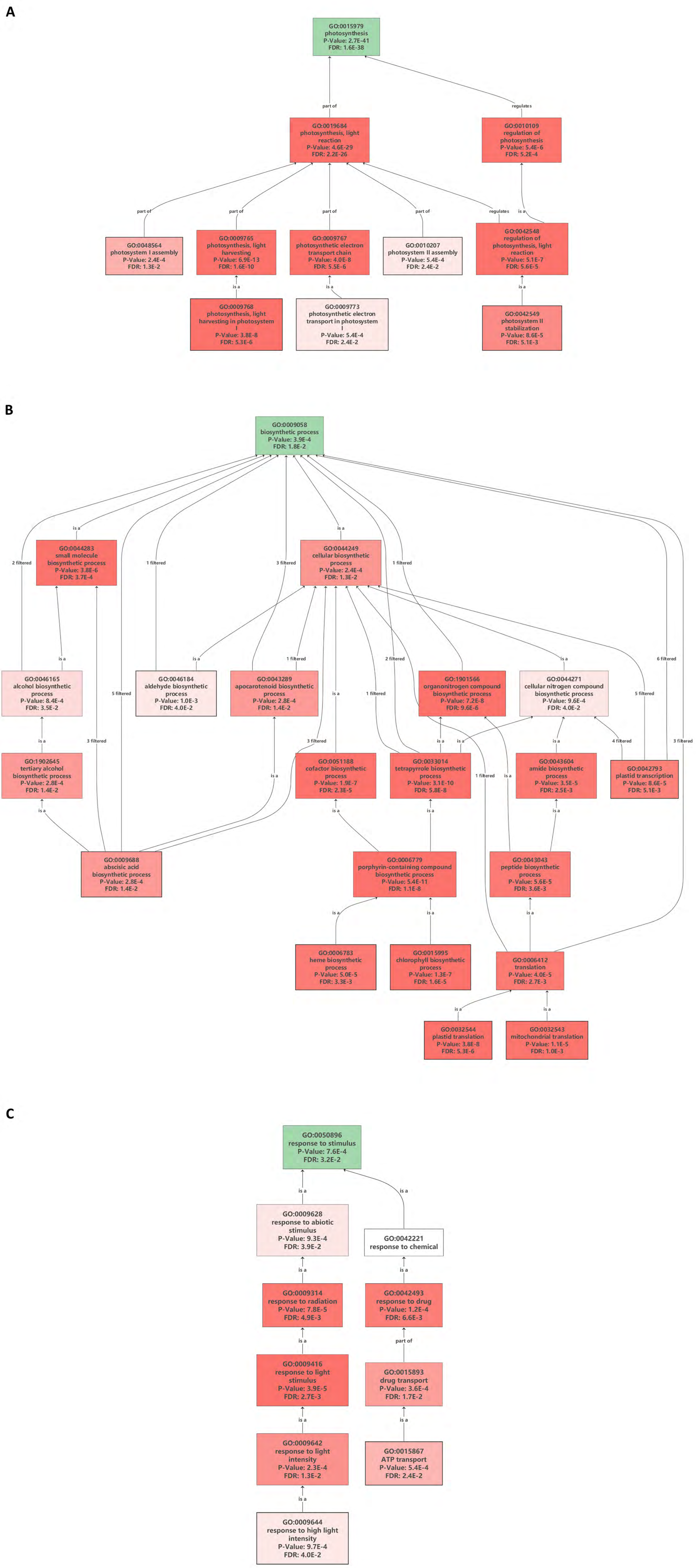


**Supplemental Figure 4. GO maps on which upregulated genes in TL compared with W were significantly enriched.**

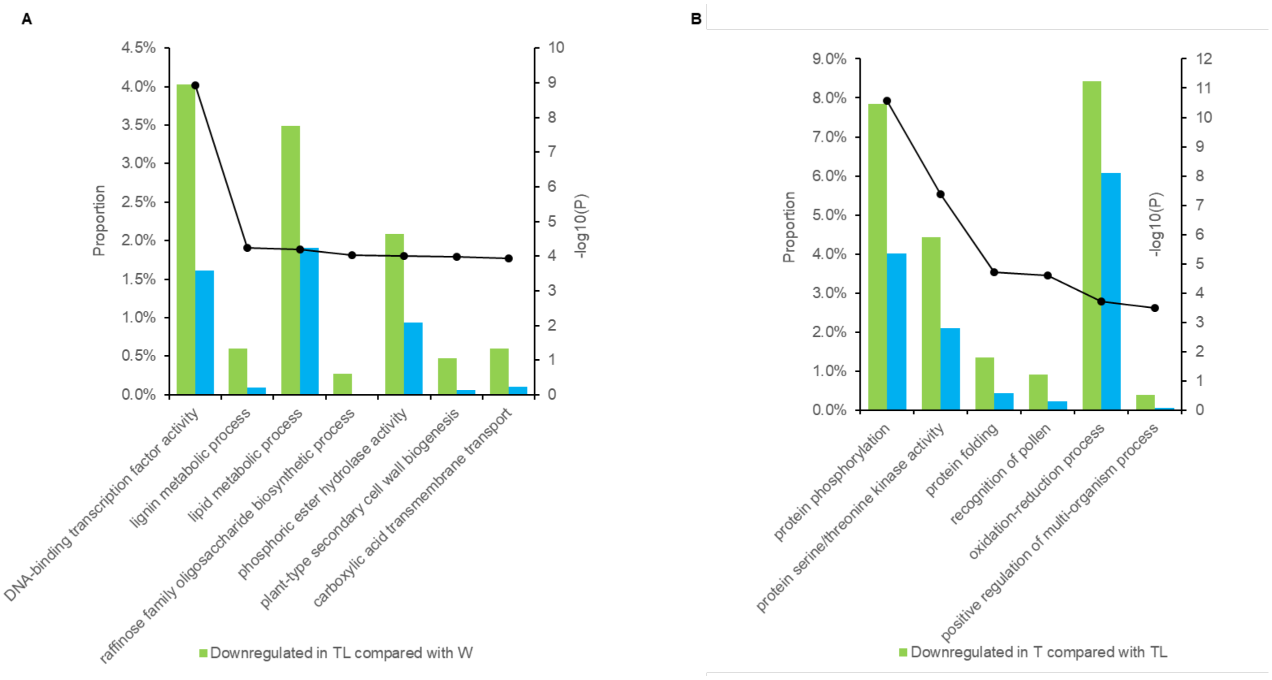


**Supplemental Figure 5. Enriched gene ontologies (GO) for downregulated genes in the two mutants.** Significantly enriched GOs in genes downregulated in TL compared with W (A) and those downregulated in T compared with TL. The bars denote the proportion of genes (the left vertical axis) with each GO in the tested gene sets, and the dots on the line denote the -log10(p-value of enrichment test) (the right vertical axis) of each GO.

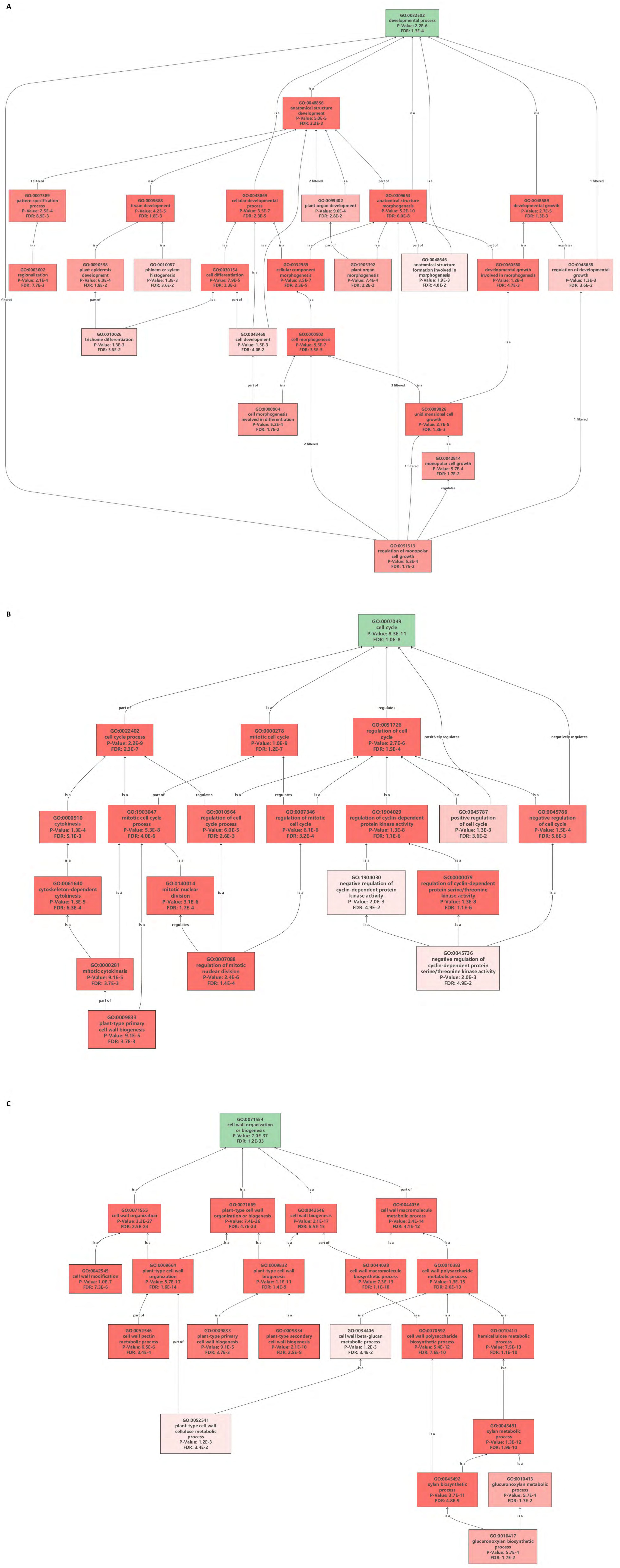


**Supplemental Figure 6. GO maps on which upregulated genes in T compared with TL were significantly enriched.**

A

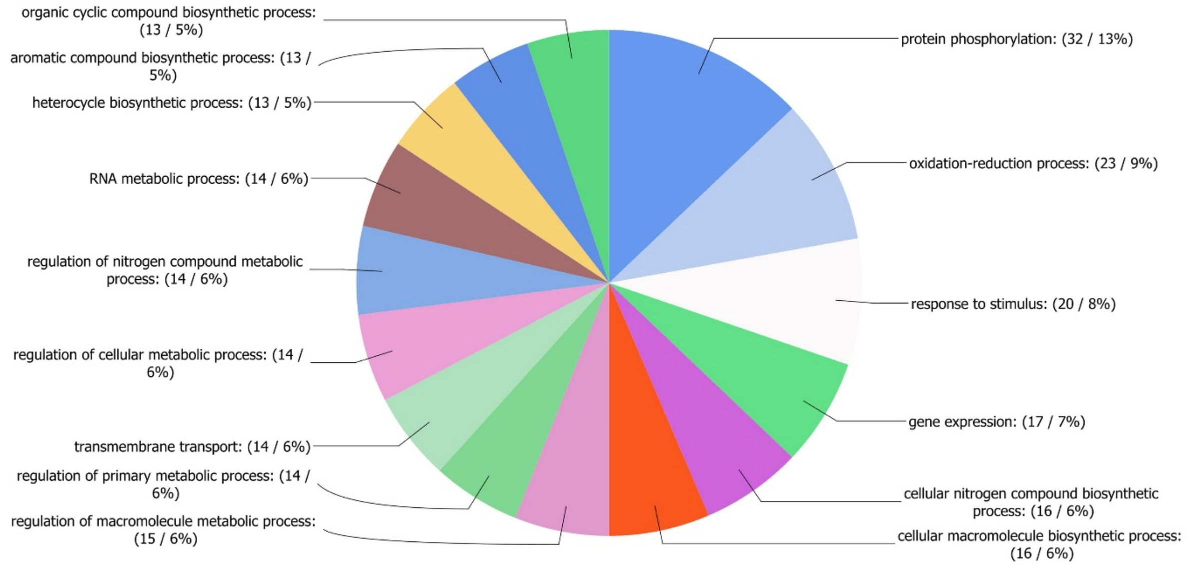


B

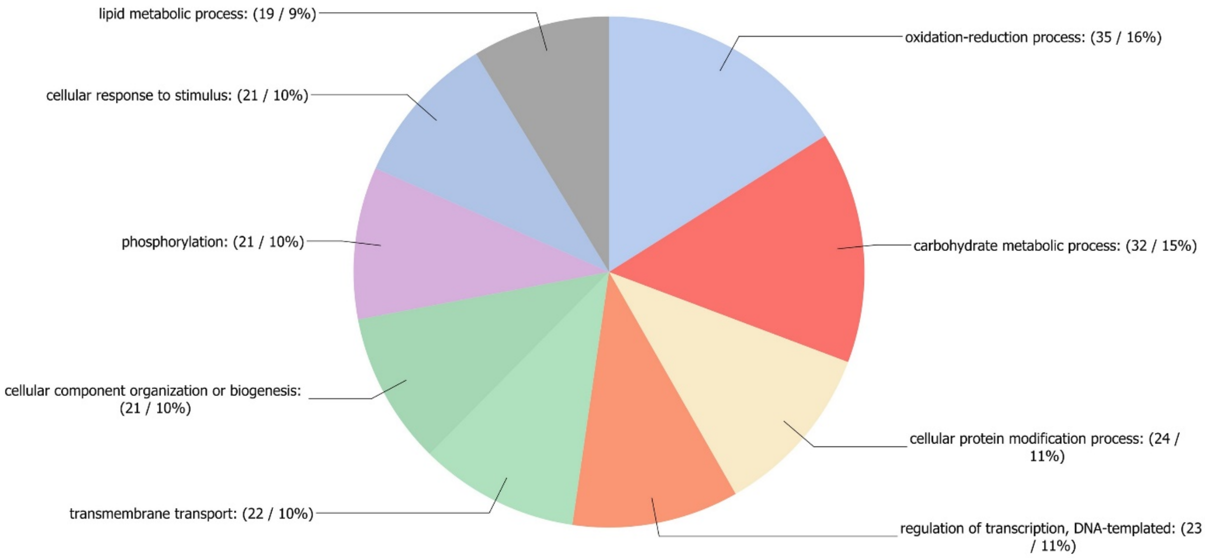


**Supplemental Figure 7. Distribution of genes upregulated (A) and downregulated (B) in TL compared with both W and T in different functional categories.**

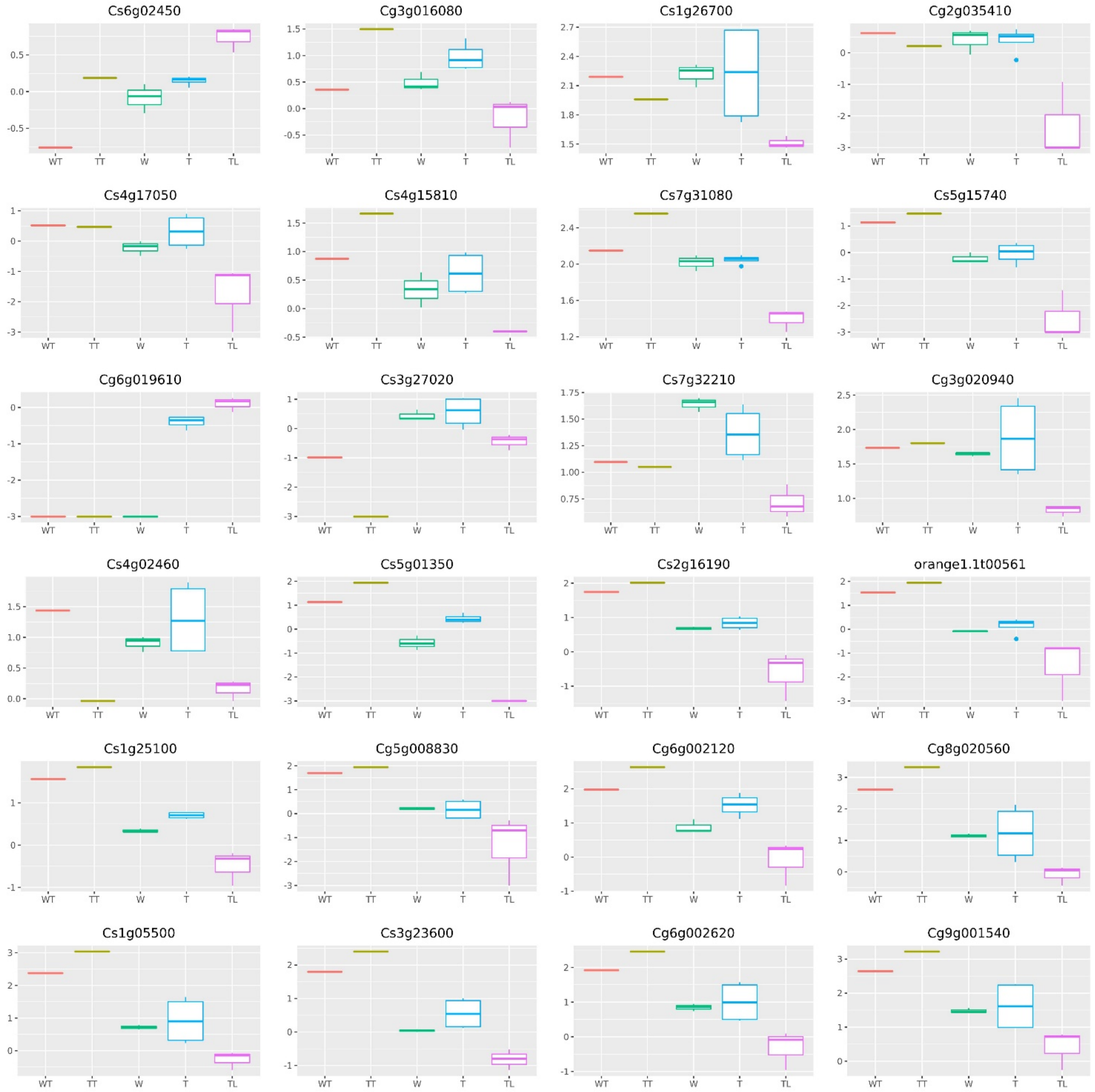


**Supplemental Figure 8. Expression of candidate genes responsible for TL-specific phenotypes.** The vertical axis denotes the log10(normalized expression value) in all the plots.

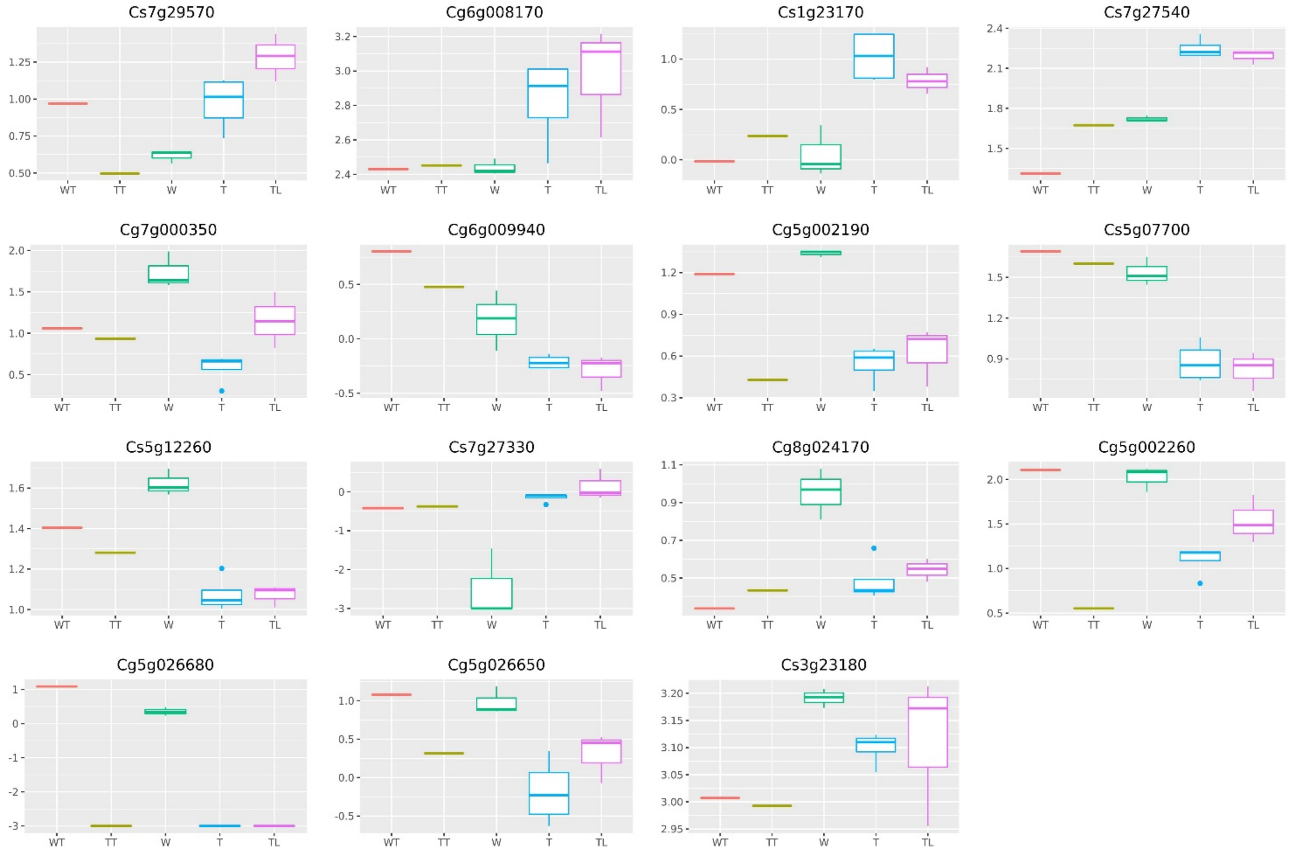


**Supplemental Figure 9. Expression of genes involved in plant defense response.** The vertical axis denotes the log10(normalized expression value) in all the plots.

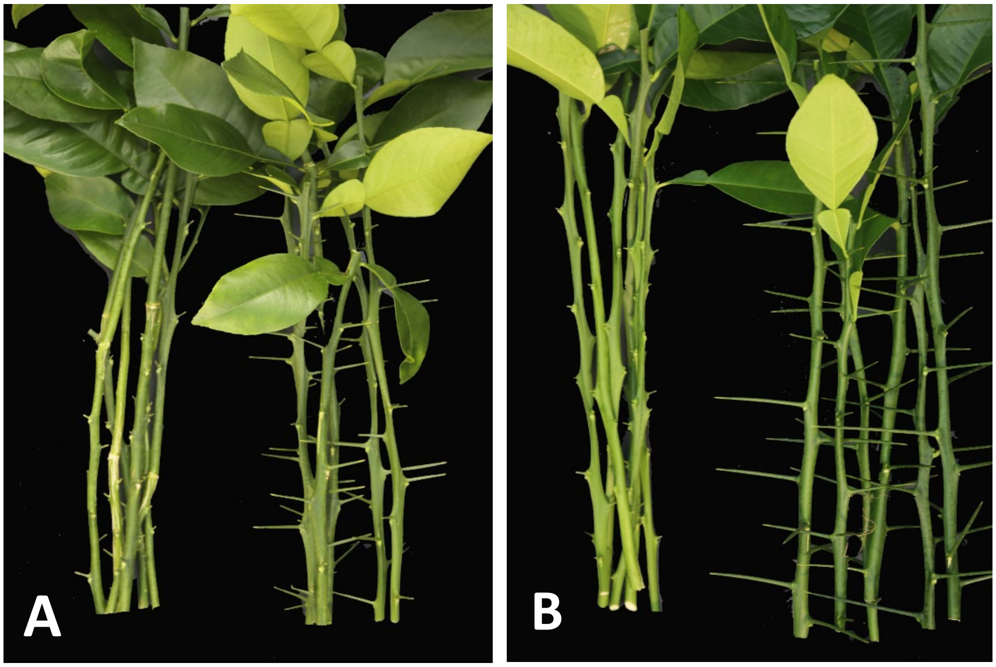


**Supplemental Figure 10. Thornless or short-thorn bud sports derived from different Citrus genotypes.** Bud sports selected from HLB-tolerant seedlings that display thron-less or short-thorn phenotypes (on the left of both pictures): A, Duncan grapefruit; B, sour orange.

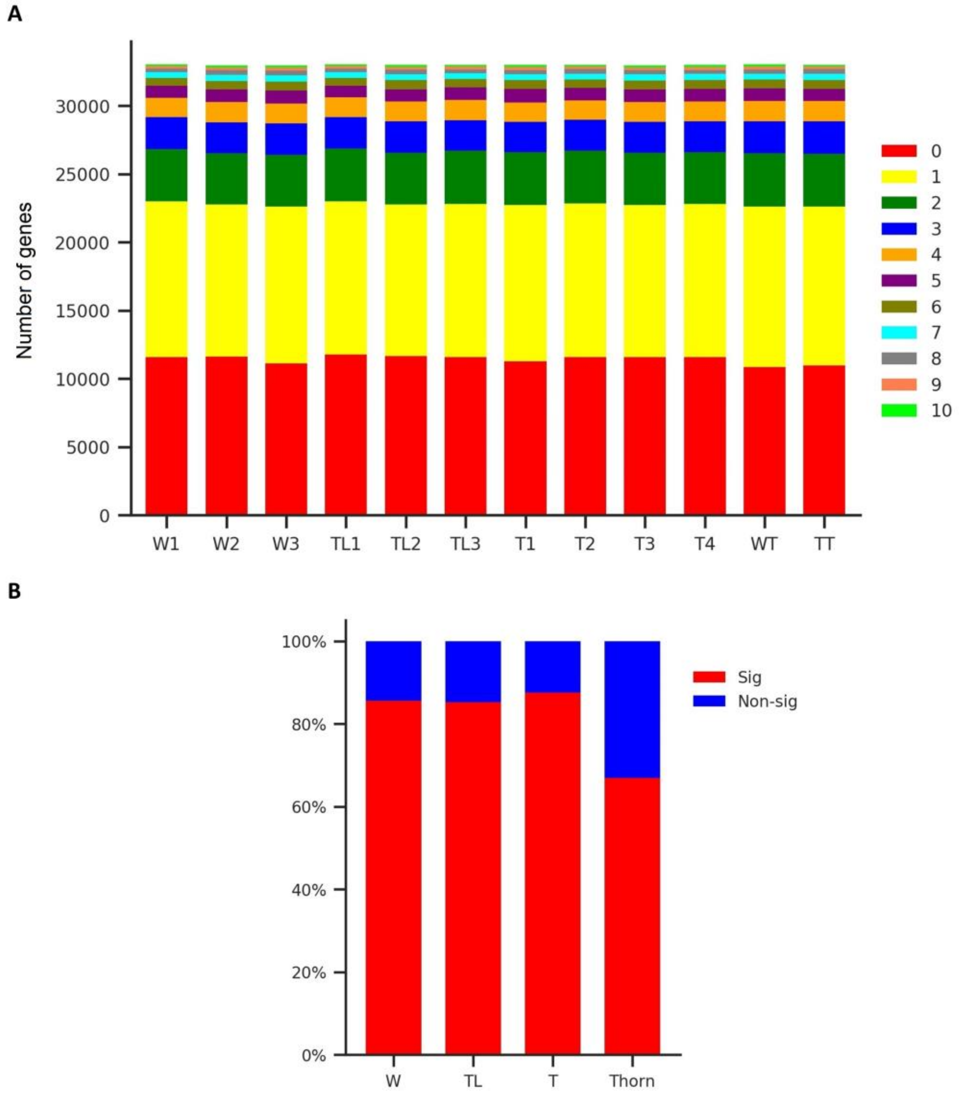


**Supplemental Figure 11. Statistics of gene isoforms in the pummelo transcriptomes.** A. Distribution of genes with different numbers of isoforms (transcripts) expressed in each transcriptome. The numbers in the legend denote the number of different isoforms detected in a transcriptome. B. Proportion of transcripts with (Sig) and without (Non-sig) significantly different expression ratio within each group.

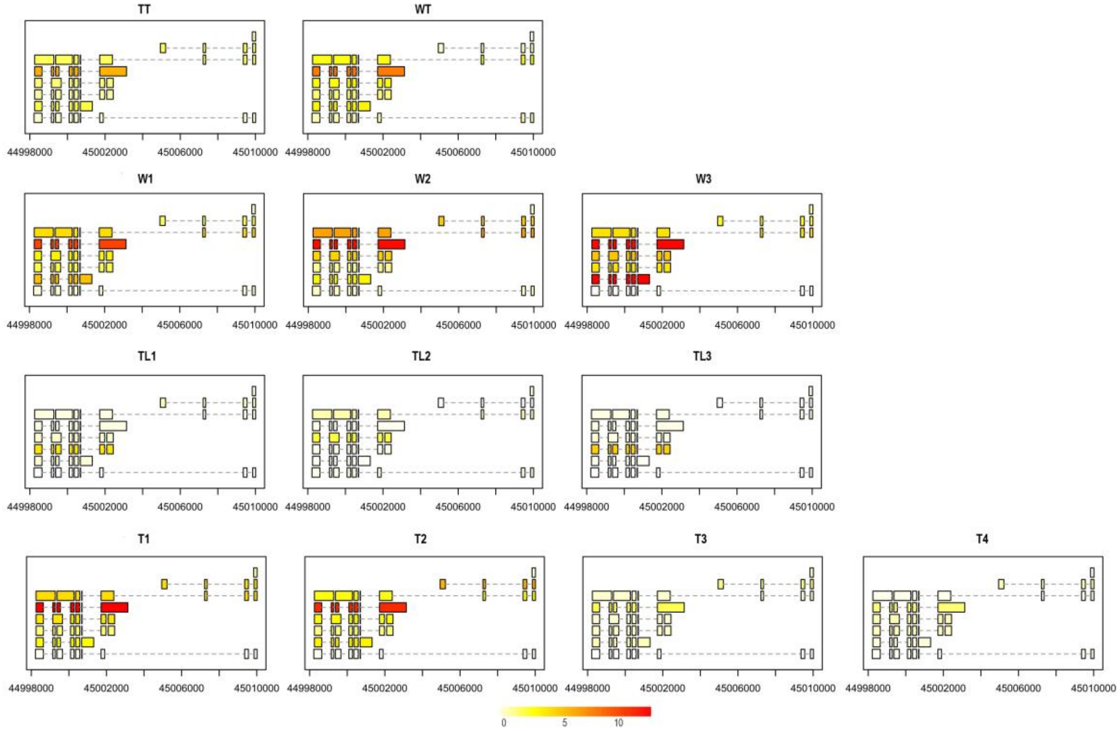


**Supplemental Figure 12. Expression of *BAT1* at the transcript level in the 12 transcriptomes.**
